# An Energy Harvesting Underwater Acoustic Transmitter for Aquatic Animals

**DOI:** 10.1038/srep33804

**Published:** 2016-09-20

**Authors:** Huidong Li, Chuan Tian, Jun Lu, Mitchell J. Myjak, Jayson J. Martinez, Richard S. Brown, Zhiqun Daniel Deng

**Affiliations:** 1 Pacific Northwest National Laboratory, PO Box 999, WA 99352, USA

## Abstract

Acoustic telemetry is the primary method to actively track aquatic animals for behavioral studies. However, the small storage capacities of the batteries used in the transmitters limit the time that the implanted animals can be studied. In this research, we developed and implemented a battery-free acoustic transmitter that uses a flexible piezoelectric beam to harvest energy from fish swimming as the power source. The transmitter sends out a unique identification code with a sufficiently strong signal (150 dB, ref: 1 μPa at 1 meter) that has a detection range of up to 100 meters. Two prototypes, 100 mm and 77 mm long, respectively, weighing only about 1 gram or less in air, were sub-dermally implanted in two species of live fish. Transmissions were successfully detected as the fish swam in a natural manner. This represents the first known implanted energy-harvesting transmitter demonstrated *in vivo*. Successful development of this transmitter greatly expands the potential for long-term studies of the behaviors of aquatic animals and for subsequently developing strategies to mitigate the environmental impacts of renewable energy systems.

Despite many years of research on salmon recovery, information across the life stages of both Atlantic and Pacific salmon, as well as other fish, is still lacking^1^. In addition, there is a critical and urgent need for a long-life monitoring technology for many other species with long life spans—including American eel and lamprey that already are^2^ or are likely to be listed as “endangered” and will have major impacts on hydropower operations^3^,^4^,^5^. Acoustic and radio telemetry have been the best available technologies for monitoring fish movement and survival in the past several decades^6^,^7^,^8^,^9^. With both technologies, a small transmitter is surgically implanted in the fish so the movement of the fish can be tracked by receivers. However, these autonomous electronic devices are limited by the finite energy capacities of their batteries. For example, one of the newest acoustic micro-transmitter for fish lasts only about 100 days, even though the battery accounts for about half of the device’s weight and volume^7^. Therefore, a transmitter that can operate without solely relying on battery power would be highly desirable. As all aquatic animals bend their bodies in certain ways to achieve spatial movements, it is conceivable that the mechanical energy generated from this repetitive motion could be exploited to provide power for small electronic devices that are either implanted or mounted on their bodies. A transmitter that can harvest this energy to power itself could greatly extend the service life or even enable self-sustainability that would allow it to operate over the lifetime of the animal being monitored.

In the last two decades, piezoelectric materials and devices have received considerable attention in energy-harvesting applications, as they provide higher energy density and higher flexibility for integration into a system when compared to electromagnetic and electrostatic devices^10^. Several studies have used piezoelectric elements to harvest mechanical energy from animal, insect, or human hosts^11^,^12^,^13^,^14^,^15^,^16^,^17^,^18^. However, the prototype devices demonstrated in those studies were either mounted on the outside of the host’s body^11^,^12^,^13^,^14^,^17^ or on the surface of the hosts’ internal organs with electrical wires connected to components outside of the host’s body^15^,^16^, which might not be practical in many applications. For example, for animal tracking applications, a device mounted on the outside of the host’s body is more likely to impede natural movement and also increase the probability of the host being exposed to predators, which could consequently bias study results. Therefore, for animal tracking applications, implantable transmitters typically are preferred. Additionally, the existing solutions are often relatively bulky and heavy as many use stacks of piezoelectric ceramic materials to achieve sufficient power conversion^17^,^18^. On the other hand, for fish, implanted devices are required to be as small and light as possible to minimize the burden that the devices impose on the fish, as a larger burden may affect the fish’s behavior and increase mortality rates^7^,^19^,^20^. Most recently, Lu *et al.*^21^ reported preliminary work of an implantable ultra-flexible energy harvester that harvests the mechanical energy from cardiac motions of a swine. In that study, only a small LED was used to demonstrate the feasibility of the device, and only the voltage output of the harvester was reported.

In this work, we developed an implantable self-powered acoustic transmitter that uses a piezoelectric composite beam implanted sub-dermally to harvest the mechanical energy from the swimming motion of fish. The piezoelectric composite beam used in this transmitter is thin, flexible and light-weight, which allows for a low-profile device that can effectively minimize the tag burden on the implanted fish. The prototypes of this transmitter were tested in a live rainbow trout (Oncorhynchus mykiss) and a white sturgeon (Acipenser transmontanus). Transmissions from transmitters were successfully detected as both fish swam naturally inside a circular tank. The output power of the energy-harvesting element was also quantitatively evaluated using a benchtop setup that replicated the prototypes implanted in the fish. To the best of our knowledge, this represents the first successful *in vivo* demonstration of an implantable energy-harvesting transmitter for host animals.

## Results

### Feasibility assessment

Prior to the actual prototyping work of this study, the feasibility of the self-powered acoustic transmitter was evaluated first by estimating the level of power output a piezoelectric energy-harvesting element could provide. Because of the anatomy of fish, a beam or strip is the most suitable geometry for an energy harvester to take advantage of the bending motions of fish swimming while minimizing the device size. Currently, there are several commercially available piezoelectric beam products that can be used for energy-harvesting applications. These products are made from piezoelectric ceramics (e.g., Volture^®^ by MIDE), polymers (e.g., the LDT series of the piezoelectric film sensors by Measurement Specialties), or composites (e.g., the Piezoelectric Fiber Composite by Advanced Cerametrics and the Macro Fiber Composite by Smart Material). In addition to the piezoelectric performance, the flexibility and the maximum strain limit of the beam are also the primary factors to consider. The Macro Fiber Composite (MFC) beams were selected for this study because they offer good balance between the flexibility needed to accommodate bending when a fish swims and piezoelectric energy conversion capability.

The feasibility of the self-powered transmitter was assessed by estimating the amount of power that could be harvested from bending the MFC beam against the power consumption of the transmitter. The active layer of a P2-type MFC beam is essentially a bundle of rectangular PZT (lead zirconate titanate) ceramic rods (or fibers) with structural epoxy surrounding them to inhibit crack propagation during bending. This configuration uses the d_31_ effect of the PZT rods to convert mechanical energy from the beam bending into electricity. In other words, the PZT rods are polarized along the thickness direction of the beam (direction “3”). However, during bending, strain is induced along the rods’ length direction (direction “1”), which is perpendicular to direction “3”. Therefore, the energy-harvesting performance of the P2-type MFC beam can be estimated by treating these rods as a monolithic piece of PZT ceramic sheet that has the same active area. Under open circuit conditions, the generated energy from a single bending motion is stored in the PZT as static electrical energy, which can be calculated by:


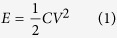


where *C* is the capacitance of the PZT and *V* is the open circuit voltage from the bending. *C* and *V* can be calculated using the Equations (2) and (3), respectively:


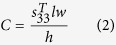



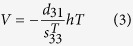


where *d*_31_ is the piezoelectric charge constant of the PZT, 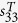 is the permittivity of the PZT, and *T* is the stress that the PZT experiences during bending. *l*, *w* and *h* are the length, width and thickness of the active area of the beam, respectively. *T* can be calculated from the bending strain, *δ*, and the tensile modulus of the PZT (*Y*):


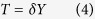


By substituting Equations (2), (3) and (4) into Equation (1), we have


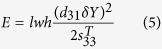


Assuming that the MFC beam bends with a bending radius of 10 cm, the maximum strain experienced by the beam is estimated to be approximately 900 ppm, which is well below the operational tensile strain limit of the MFC (4500 ppm)^22^. To estimate the energy conversion capability of a P2-type MFC beam under bending strain, one can assume that the entire beam experiences an average strain of half the maximum strain experienced at the center of the beam. For a P2-type MFC beam (Model 8503) that has an active area of 85 × 3 mm^2^ and a PZT-5A fiber thickness of 0.18 mm, inputting the actual values of the parameters in the equation, we show that *V* = 18 volts and *E* = 4.3 μJ. Therefore, if the tail-beat frequency of the implanted fish is 1 Hz (i.e., the beam bends twice per second), the generated power from the beam would be 8.6 μW.

For comparison, the energy consumption of a single 150-dB (reference: 1 μPa at 1 m) transmission for the injectable acoustic transmitter that we recently developed was measured to be 12 μJ. If a typical ping rate of 3 seconds is used, the power requirement of the transmitter would be 4 μW, less than the generated power from the beam at 1 Hz. Therefore, we determined that it would be feasible for an 8503 MFC beam to power our transmitter at a 150-dB source level.

### Transmitter design

**Figure 1:**
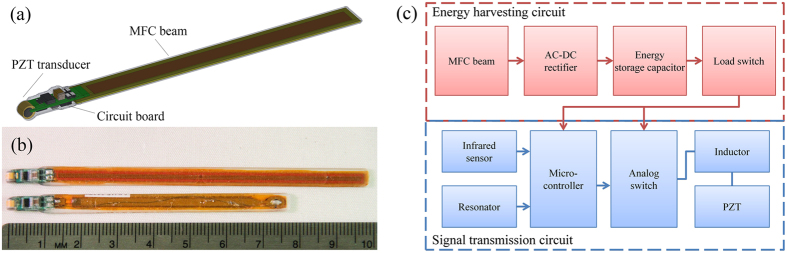
Design of the self-powered acoustic transmitter: (**a**) CAD of the transmitter; (**b**) photograph of the actual transmitter prototypes; (**c**) circuit block diagram of the transmitter.

The self-powered transmitter consists of three main components (Fig. 1a): 1) a PZT-5H tube transducer that transmits acoustic signals, 2) a circuit board that contains both the transmission circuit and the energy-harvesting circuit, and 3) a MFC beam as the energy-harvesting element, which is the sole power source of the entire transmitter. The transmitter is 5.3 mm wide but only 1 mm thick for most of its body length (the MFC portion). The transducer and the circuit board constitute the thickest portion of the transmitter body, which is 3.0 mm thick but accounts for only a small portion of the total length. The transmitter is encapsulated in clear urethane rubber and finished with a 25-μm-thick Parylene-C protective layer for waterproofing and biocompatibility. Two prototypes using the 8503 and 5003 (50 × 3 mm^2^ active area) MFC beams, respectively, were fabricated and tested in this study (Fig. 1b). The prototype using the 8503 MFC beam was 100 mm long, 0.57 cm^3^ in volume, and weighed 1.05 grams in air. The shorter prototype using the 5003 MFC was 77 mm long, 0.44 cm^3^ in volume, and weighed 0.80 grams in air.

### Circuit design

The circuit design of the self-powered transmitter (Fig. 1c) includes two sub-circuits that handle two distinct functions: 1) energy harvesting and 2) signal transmission. The energy-harvesting sub-circuit consists of an AC-DC rectifier, a 22-μF capacitor for energy storage, and a special load switch with both switch-on and switch-off voltages. The rectifier converts the AC output voltage from the MFC beam to DC voltage, which charges the storage capacitor. The energy loss through the self discharge rate of the capacitor (3.5 μW) is slower than energy harvesting power of both the 8503 and 5003 MFC bending at 1 Hz (8.6 and 5.2 μW, respectively). The load switch is used to interface between the storage capacitor and the transmission sub-circuit (i.e., the load). It has a switch-on voltage (V_on_) of 2.8 Vdc and a switch-off voltage (V_off_) of 2.3 Vdc. When the switch senses that the voltage of the storage capacitor is higher than V_on_, it automatically turns on so the transmission sub-circuit can draw the stored energy in the capacitor and start transmitting at a set ping rate. The switch will stay on to enable transmission until the capacitor voltage drops below V_off_, at which point the switch shuts off and the transmitter completes a “bundle” of transmissions. The transmission sub-circuit will then stay in this “off” mode until the storage capacitor is charged to a voltage level higher than V_on_ again. Thus, the time span between the transmission bundles can be considered the recovery period of the transmitter.

Using of a load switch with both switch-on and switch-off voltages was a design decision that took into account multiple factors. First, these two threshold voltages provide the transmitter a voltage window for operation. This window allows regulation of the amount of energy that can be drawn from the storage capacitor. Second, the relatively high V_off_ (the minimum V_off_ allowed by the load switch is 1.7 V) prevents the storage capacitor from being drained to such a low voltage level that would require the implanted fish to swim continuously for a much longer period of time before transmissions could start again (i.e., a longer recovery period). Because of the intrinsic self-discharge of the storage capacitor, if the implanted fish swims only for a short period of time and goes into rest before the capacitor voltage level reaches the V_on_, the accrued energy from the brief swimming period will become partially or even completely lost during the fish’s rest. A long recovery period would increase the likelihood of the occurrence of this scenario, leading to an overall lower energy-harvesting efficiency and less frequent signal transmissions. Therefore, the relatively high V_off_ helps shorten the recovery period and also enables more frequent transmission.

The prototype transmitter was designed to make multiple transmissions of the same identification code with a preset ping rate so the transmitted identification code can be positively identified. The nominal signal strength of the transmitter is 150 dB (reference: 1 μPa at 1 m), which allows a detection range of up to 100 m in fresh water. Based on our measurements, the power consumption at this source level was 12 μJ. Therefore, the capacitance (22 μF) of the storage capacitor and the threshold voltage window of the load switch were carefully selected to allow at least two transmissions to be made before the capacitor voltage decreased to a level below V_off_.

The transmission sub-circuit was modified from the injectable acoustic transmitter for the Juvenile Salmon Acoustic Telemetry System (JSATS) developed previously^7^. An ultra-low-power microcontroller unit is used to control an analog switch that generates the coded voltage signals needed to drive the PZT transducer. A ceramic resonator at the input of the microcontroller unit generates a precise clock signal that controls the modulation frequency of 416.7 kHz. To improve efficiency, a tuning inductor is connected in series with the PZT to establish an electrical resonance near 416.7 kHz.

### Benchtop experiment

**Figure 2:**
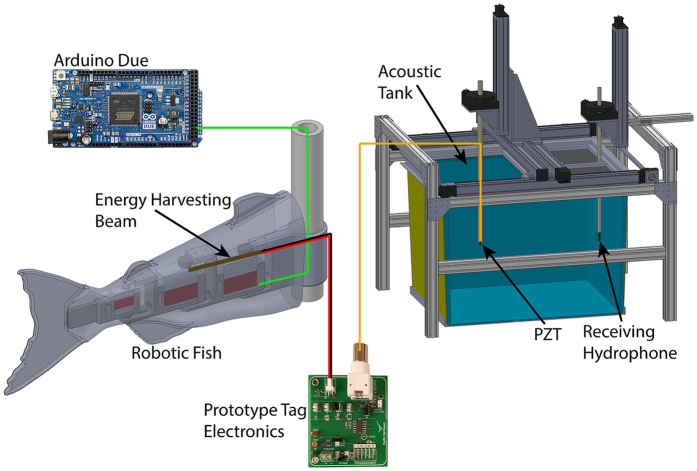
Schematic of the benchtop experiment setup.

The actual prototype transmitter would be entirely encapsulated in urethane rubber and implanted into a live fish; therefore, it would have no wire connections for electrical measurements. To quantitatively investigate the energy-harvesting performance of the transmitter, the following components were used on the benchtop (Fig. 2) to replicate those in the actual prototype:

- MFC beams backed with a 75-μm-thick aluminum shim and coated with the same urethane rubber as that in the actual transmitter
- A robotic fish as described below in the Methods section to simulate bending motions
- A test circuit board containing the identical circuitry as that used in the prototype
- A PZT tube (same as used in the prototype) that was waterproofed by epoxy for underwater signal transmission. Its inner and outer wall electrodes were connected to the circuit board via a shielded coaxial cable.

Similar to a real fish, the bending radius of the robotic fish body may not be consistent throughout the entire body length and hence may lead to variations in the output power for different implantation locations. The selected implantation location was on the back of the robotic fish under the dorsal fin and the amount of bending of the fish body was carefully configured so the bending radius was as close to 10 cm as possible.

**Figure 3:**
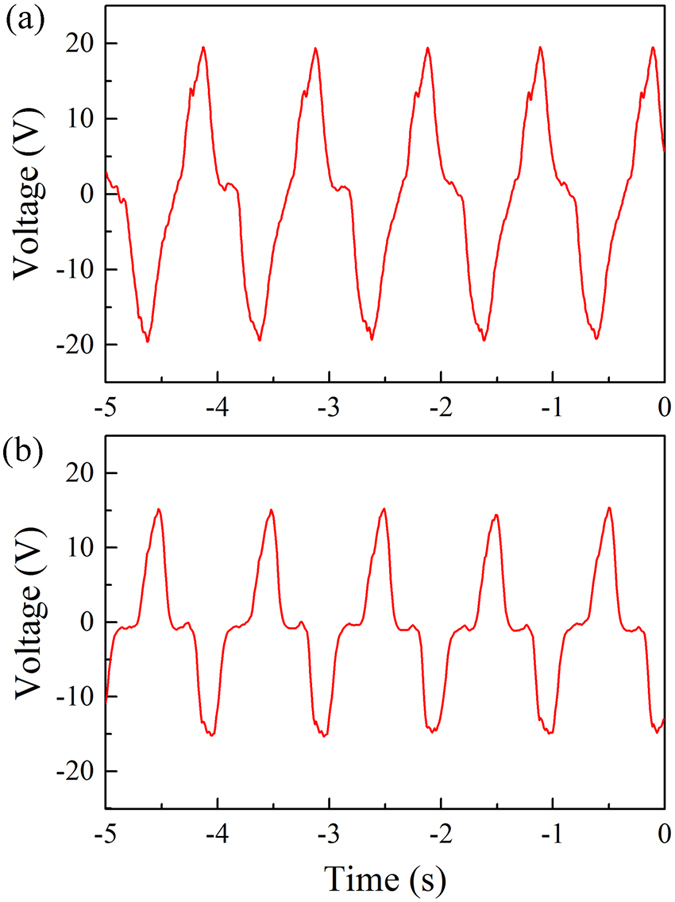
Open-circuit voltage outputs of the 8503 and 5003 MFC beams at 1 Hz: (**a**) 8503 MFC beam; (**b**) 5003 MFC beam.

The open circuit voltages of the 8503 and 5003 MFC beams at a tail-beat frequency of 1 Hz are shown in Fig. 3. The 8503 beam output ~19 V in amplitude, consistent with the theoretical calculation results obtained in the feasibility assessment. The 5003 beam, on the other hand, output ~16 V in amplitude, slightly lower than that of the 8503 beam. According to Equation (3), the output voltage of the beam is only related to the thickness and intrinsic material properties of the PZT. Since both beams had the same thickness, this difference in the output voltage indicates that the longer length of the 8503 beam allowed it to more readily conform to the fish’s body, hence incurring slightly higher strain, as the fish bent.

**Figure 4:**
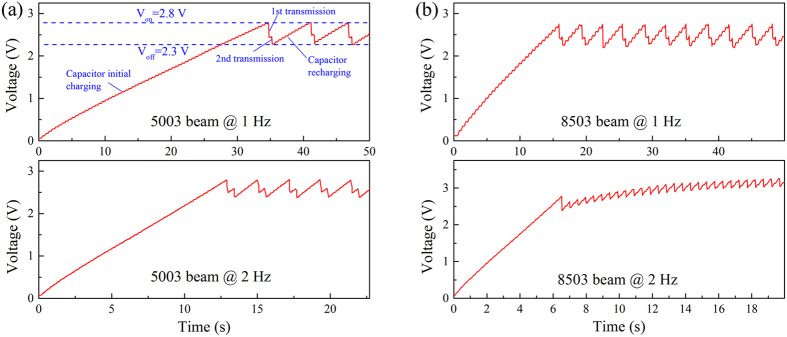
Voltage of the storage capacitor as a function of time during operation at 1 Hz and 2 Hz: (**a**) 5003 MFC beam; (**b**) 8503 MFC beam.

To evaluate the energy-harvesting performance, the voltage of the 22-μF storage capacitor as a function of time at tail-beat frequencies of 1 Hz and 2 Hz were measured (Fig. 4). In this experiment, the transmitter was programmed to transmit at a pulse rate interval of 0.5 seconds once the capacitor voltage reached V_on_. The voltage first ramped up continuously from zero as the capacitor was charged by fish bending. As the voltage reached the V_on_, the transmitter started to draw energy from the capacitor, and transmissions began. The voltage of the capacitor then dropped abruptly. The length of each JSATS signal was 0.744 ms. The V_on_ (2.8 V) and V_off_ (2.3 V) of the switch dictated that the 22-μF capacitor would release about 28 μJ of energy to the transmission sub-circuit before the switch shut off. Each transmission consumed about 12 μJ, which was estimated by measuring the current draw of the transmission circuit through a 10-ohm resistor when the transmission circuit was driven by a DC power supply outputting 2.5 V. Therefore, as the capacitor voltage reached V_on_, the capacitor contained energy sufficient to make at least two transmissions before its voltage dropped below V_off_. Figure 4 shows that when the first transmission ended, the capacitor voltage dropped to about 2.4 V and the voltage increased again as the robotic fish’s swimming motion continued. Half a second after the first transmission, a second transmission occurred as programmed, and the capacitor voltage dropped again and reached V_off_ (except for the 8503 beam at 2 Hz), at which point the switch turned off and the transmitter hence completed a transmission “bundle.” From this point on, for the transmitter to transmit again, the capacitor would need to be recharged to 2.8 V.

If the power loss due to the self-discharge of the capacitor is ignored, the power harvested by the energy harvesting unit can simply be quantified using the 28-μJ energy released by the capacitor and the recovery time between the transmission bundles. At 1 Hz, it took the 8503 and 5003 beams 16 and 35 seconds, respectively, to charge the storage capacitor from a completely drained state (0 V) to 2.8 V, and the increase of the capacitor voltage was nearly linear. As the fish continued the swimming motion at the same frequency, the transmission bundles were able to maintain a constant interval of 3 seconds for the 8503 beam and 6 seconds for the 5003 beam, which corresponded to energy-harvesting powers of 9.3 μW and 4.7 μW, respectively. These values were consistent with the aforementioned feasibility assessment. At 2 Hz, the capacitor was charged to 2.8 V much more quickly. It took the 8503 and 5003 beams merely 6.5 and 13 seconds, respectively, to start the first transmission. The 5003 beam was able to maintain a constant interval of about 2 seconds between the transmission bundles as the robotic fish continued to “swim” at that frequency, which corresponded to energy-harvesting power of 14 μW. On the other hand, for the 8503 beam, power input to the transmitter was so ample that not only could the transmitter maintain continuous transmission at the set 0.5-second pulse rate interval but the overall voltage level of the capacitor slowly increased over time, which indicated an energy-harvesting power greater than 24 μW.

### Live fish experiment

**Figure 5:**
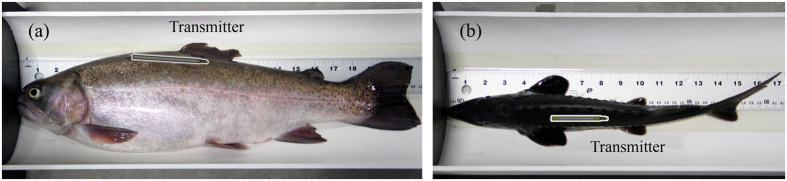
Photographs of fish implantation: (**a**) implantation location of the 100-mm-long transmitter for the rainbow trout; (**b**) implantation location of the 77-mm-long transmitter for the white sturgeon.

On October 22, 2015, the prototype transmitters (Fig. 1b) using the 8503 and 5003 MFC beams, respectively, were implanted and tested in live fish. The 100-mm transmitter was implanted in a 53-cm (fork length) rainbow trout and the 77-mm transmitter was implanted into a 38-cm (fork length) white sturgeon (Fig. 5). After the implantation, both fish were placed into a circular tank (diameter: 1.2 meters) that was installed with two hydrophones for listening to the transmitted acoustic signals.

**Figure 6:**
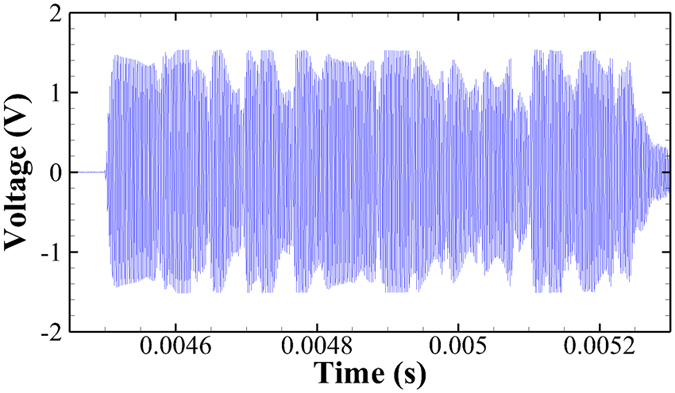
Acoustic waveform received from the self-powered transmitter implanted in the white sturgeon.

Both fish recovered from anesthesia and started transmitting coded acoustic signals just several minutes after being released into the circular tank. The individual signal waveforms from the two transmitters were successfully detected by the JSATS detection software (Fig. 6). The waveforms were the amplified output voltages of the hydrophones as functions of time. Each waveform was a 744-μs-long signal that carried a unique JSATS 31-bit tag code, which includes a 7-bit header, a 16-bit identification number, and an 8-bit cyclic redundancy check^23^. The fish were monitored in the tank for two weeks. Throughout the course of the experiment, neither fish showed apparent signs that their swimming motions were inhibited by the transmitter. The transmitter implanted in the juvenile sturgeon made more than 100 transmissions per hour, much more frequent than the transmissions from the trout (0–6 transmissions per hour), although the MFC beam implanted in the sturgeon was the shorter one. Based on our observations, the transmissions from the trout were less frequent possibly because the implantation location on the trout was a bit too forward relative to its body length, which resulted in a much lower degree of beam bending as the fish swam. Another reason could be that the circular tank was relatively small for the size of the trout so its swimming activity was limited. On the other hand, the smaller sturgeon might have needed to swim faster to keep itself stationary against the circulating water flow inside the tank. Its tail-beat frequency was observed to be mostly around 2 Hz, while the trout’s swimming behavior was much more erratic and the degree of body bending was much less most of the time when compared to the sturgeon.

## Discussion

This research successfully developed and demonstrated battery-free acoustic transmitters that can harvest energy from the swimming motion of an aquatic animal to power a transmitter. The energy-harvesting performance also was characterized on a benchtop setup using a robotic fish. We showed that the power outputs of the two energy-harvesting prototypes were 9.3 μW and 4.7 μW, respectively, at 1 Hz, and 14 μW and >24 μW at 2 Hz.

The design of the transmitter may be modified to meet requirements of specific active tracking applications. The actual size of the transmitter is primarily dictated by the size of the MFC beam used, which is ultimately dependent upon the physiology and swimming behavior of the specific species of the animal as well as the requirement of the signal strength. While ensuring that the power requirements are met, the thickness of the MFC beams or metal shims could be varied for different flexibility requirements. However, to prevent transmitter failure, it is important to note that the maximum bending strain should not be close to or exceed the maximum strain limit of the MFC. For higher power requirements, additional MFC beams could be added, or a rechargeable battery could be incorporated so the transmitter could operate constantly regardless of the host animal’s activity level.

Evidently, further investigation is still necessary to practically use these transmitters in a field environment. Tag effect studies will need to be conducted to determine the appropriate sizes of the animal that can be implanted for the transmitters that use different sizes of the MFC. Work still needs to be done to ensure the reliability of the energy harvesting beam and the operation of the transmitter under high pressure. We do not expect water temperature to significantly affect the energy harvesting performance as the piezoelectric and mechanical properties of PZT-5A material used in the MFCs are relatively stable in the temperature range of 0 to 30 °C.

This is the first reported demonstration of a viable energy-harvesting technology that can be implanted in live aquatic animals to provide power for micro electronic devices without inhibiting the host animal’s natural movements. Successful development of the self-powered transmitter and its energy-harvesting unit will enable researchers to conduct long-term monitoring of animals. For example, this technology could make it possible to understand the long-term environmental impacts of renewable energy systems such as hydroelectric and marine power systems. The ability to actively monitor fish over several years could entirely change the way that fish research is conducted. Furthermore, it also is conceivable that the energy-harvesting technology developed in this research could also be used in other applications where micro-electronic devices can benefit from the mechanical energy available from animal movements.

## Methods

### Prototype fabrication

The prototype transmitters used a P2-type piezoelectric MFC beam (Smart Material Corp., Sarasota, Florida, USA) as the energy-harvesting element. The MFC consists of rectangular PZT-5A ceramic rods sandwiched between layers of adhesives, electrodes, and polyimide film^22^. The active material area of the MFC beams used in the prototypes was 3 mm wide. To target fish of different sizes, prototypes of two different lengths, 100 mm and 77 mm, were developed using M-8503-P2 (active material area: 85 × 3 mm^2^) and M-5003-P2 (active material area: 50 × 3 mm^2^) MFC beams, respectively. Because a lone MFC beam under bending would theoretically have minimal electricity output due to the opposite signs of stress experienced by the top half and bottom half of the beam^24^, a 75- μm thick aluminum shim was attached on the back of the MFC beam using epoxy so that the majority of the PZT material in the beam could be under stress of the same sign. In addition, it also helped to increase the beam’s fatigue resistance.

The piezoelectric transducer used in the prototype for transmitting acoustic signals was a 610HD PZT ceramic tube (TRS Technologies, State College, Pennsylvania, USA) that had an outer diameter of 2.54 mm, an inner diameter of 1.80 mm, and a length of 1.75 mm. The center of the inner circumference of the tube was offset toward the front of the transmitter by 0.15 mm from that of the outer circumference. This transducer design helps to direct more acoustic energy away from the direction where the emitted acoustic waves are blocked by the circuit board^25^. The MFC beam and the PZT transducer were attached to the circuit board by soldering and silver epoxy, respectively. The transmitter assembly was first coated with a 25-μm thick Parylene-C layer and then encapsulated with ClearFlex 95 urethane rubber (Smooth-On Inc., East Texas, Pennsylvania, USA). Another 25-μm layer of Parylene-C was deposited on the outside of the transmitter as a waterproof and biocompatible layer.

### Benchtop testing

To evaluate the energy-harvesting performance of the prototype, MFC beams with the same specifications and the same aluminum shim as those used in the prototypes were tested on a benchtop setup. These test beams also were coated with the ClearFlex 95 of the same thickness as that on the actual prototypes. To mimic the swimming motion of fish, a robotic fish was fabricated based on the three-dimensional representation of an actual Chinook salmon. It contained three interconnected servo-motors wrapped inside a soft silicone gel skin. To allow the beam to bend with the fish body, the self-powered transmitter was designed to be implanted sub-dermally instead of inside the body cavity of the fish, because the body cavity would allow too much room for the beam to relax and thus might limit the achievable amount of bending in the beam. Therefore, for the benchtop experiment, the MFC beam was inserted on the back of the robotic fish in a pocket under the dorsal fin (shown in Fig. 2) and about 1 mm underneath the surface of the faux fish. To insert the MFC beam, a pocket was created by slicing with an X-ACTO knife. The pocket was slightly longer than the beam and was about 1 mm beneath the body surface. The beam was then placed inside the pocket and sealed using EcoFlex 00–30 gel.

During testing, the robotic fish was controlled via an Arduino Due board and was driven with a tail-beat frequency of 1 or 2 Hz. The bending radius of the robotic fish body was set at approximately 10 cm to be consistent with the assumed value used in the feasibility assessment. The MFC beam was electrically connected to a test circuit board that had the same circuit as that of the actual prototypes. The output of the circuit board was connected to a PZT tube transducer that was the same as the one used in the actual transmitter prototype. The transducer was epoxy coated so it can be tested underwater for transmission performance. The output voltage of the MFC beam and the voltage of the storage capacitor that stored the harvested energy were monitored by an oscilloscope.

### Prototype implantation and live fish experiment

Implantation of the prototype transmitters and the live fish experiment were performed at the Aquatic Research Laboratory of Pacific Northwest National Laboratory. The holding conditions and all experimental procedures were approved by and carried out in accordance with the guidelines of the Institutional Animal Care and Use Committee (IACUC) of Pacific Northwest National Laboratory.

As in the robotic fish, these prototypes were implanted on the back of the fish near the dorsal fin. However, considering that the MFC’s in real fish may experience much higher degree of bending than in the robotic fish in some occasions, which may damage the PZT fibers within the MFCs, the implantation locations selected for the two test fish were in the front-half of the fish body where a much lower degree of bending occurs. Prior to implantation, fish were anesthetized with 80 mg/L of tricaine methanesulfonate (Syndel Laboratories Ltd., Qualicum Beach, British Columbia, Canada) to stage-4 anesthesia. During implantation, a 6-mm incision was first made with a scalpel that only cut barely beneath the skin. A flattened stainless steel 8-gauge needle was then used to carefully separate the skin from the muscle to open a channel that was about 2 mm longer than the transmitter length. The transmitter was then inserted into the channel with the transducer end entering first. Once the transmitter was completed inserted, the incision was sutured with one knot using 3-0 suture. The entire implantation process took about 75 seconds. After implantation, both fish were placed into a circular tank (1.2 m diameter) with two hydrophones (Model SC001, Sonic Concepts Inc., Bothell, Washington, USA) installed and filled with room-temperature river water for recovery and subsequent acoustic signal monitoring.

## Additional Information

**How to cite this article**: Li, H. *et al.* An Energy Harvesting Underwater Acoustic Transmitter for Aquatic Animals. *Sci. Rep.* **6**, 33804; doi: 10.1038/srep33804 (2016).
